# Excessive/Aberrant and Maladaptive Synaptic Plasticity: A Hypothesis for the Pathogenesis of Alzheimer’s Disease

**DOI:** 10.3389/fnagi.2022.913693

**Published:** 2022-07-05

**Authors:** Shigeki Kawabata

**Affiliations:** Dementia Project Promotion Office, Sompo Care Inc., Tokyo, Japan

**Keywords:** Alzheimer’s disease, synapse, plasticity, Amyloid - beta, APP – amyloid precursor protein, presenilin, ApoE4

## Abstract

The amyloid hypothesis for the pathogenesis of Alzheimer’s disease (AD) is widely accepted. Last year, the US Food and Drug Administration considered amyloid-β peptide (Aβ) as a surrogate biomarker and approved an anti-Aβ antibody, aducanumab, although its effectiveness in slowing the progression of AD is still uncertain. This approval has caused a great deal of controversy. Opinions are divided about whether there is enough evidence to definitely consider Aβ as a causative substance of AD. To develop this discussion constructively and to discover the most suitable therapeutic interventions in the end, an alternative persuasive hypothesis needs to emerge to better explain the facts. In this paper, I propose a hypothesis that excessive/aberrant and maladaptive synaptic plasticity is the pathophysiological basis for AD.

## Introduction

Research on Alzheimer’s disease (AD) began with the elucidation of its pathological features. Once the amyloid plaque (AP) substance was identified as amyloid-β peptide (Aβ), which is produced from amyloid β precursor protein (APP), the amyloid hypothesis became scientific orthodoxy in academia and has remained so for many years. APP is processed through two enzymatic pathways, namely non-amyloidogenic α-pathway and amyloidogenic β-pathway. Within α-pathway, the first step of the proteolysis is performed by α-secretase and the large ectodomain, so-called sAPPα, and the C-terminal fragment (CTF) which remains anchored to the membrane (CTFα) are produced. In β-pathway, β-secretase cleaves APP and generates sAPPβ and CTFβ. At the second step of the proteolysis, CTFα and CTFβ are both cleaved by γ-secretase, but Aβ is only produced from CTFβ (Carrillo-Mora et al., 2014). The striking discoveries, which moved the amyloid theory forward, are the linkage of the gene mutations in APP and presenilin, the catalytic core of γ-secretase complex (PSEN1 and PSEN2) to Familial Alzheimer’s disease (FAD). The amyloid hypothesis states that Aβ is toxic to neurons and that FAD-linked mutations in the APP and presenilin genes lead to the production of more toxic Aβ such as Aβ42 and other longer species of Aβ (Selkoe and Hardy, 2016). However, when analyzing more than a hundred types of gene mutation found in FAD, the produced Aβ was found to vary in length and amount among the mutations (Li et al., 2016; Sun et al., 2017; Devkota et al., 2021). Such variability raises the question of which types of Aβ exert toxicity. A debatable fact is that some FAD-linked PSEN1 mutations drastically reduce the production of Aβ42, a type of Aβ that have been widely studied in the context of AD causality (Sun et al., 2017). Various mechanisms of AD causation have also been reported (Carrillo-Mora et al., 2014), such as oxidative stress, glutamate toxicity as well as impairment of various signal transductions and several intracellular mechanisms. It is not well understood how Aβ acquires multiple functions, and which mechanism of action contributes to the causal neurotoxicity. Issues raised by the amyloid hypothesis are still unresolved.

In this paper, I integrate the past research findings from a different angle from what has been discussed in connection with the amyloid theory, and propose a hypothesis that excessive/aberrant and maladaptive synaptic plasticity is the cause of AD.

## The Genes Associated With Familial Alzheimer’s Disease

Research on APP and presenilin has been focused on how mutations found in FAD alter the mode of Aβ production. However, when a gene mutation is linked to the disease, questions arise including what the original function of the gene is, whether the mutation affects that function, and if so, whether the functional abnormality causes the disease. The function of APP has been elucidated by studies in mice and *Drosophila* (Torroja et al., 1999; Leyssen et al., 2005; Soldano et al., 2013; Klevanski et al., 2015). *Drosophila* has an APP homolog called APPL. These studies have shown that APP plays an important role in synaptic plasticity such as neurite extension and synaptogenesis and that the CTF, especially the cytoplasmic domain of APP is critical for the function of APP. In *Drosophila*, axonal arborization of the small ventral lateral neurons is induced by full-length APPL or the CTF only, but not by APPL lacking the intracellular region (Leyssen et al., 2005). Accumulation of membrane-tethered intracellular domain of APP in rodent neuronal cultures also results in marked increase in neurite extension (Deyts et al., 2012). The importance of APP or APPL in synaptic plasticity has also been shown in the Mushroom Bodies, a *Drosophila* center of olfactory learning (Soldano et al., 2013) and the neuromuscular junction of *Drosophila* (Torroja et al., 1999), and in the central nervous system and the neuromuscular junction of mice (Klevanski et al., 2015). Although a large ectodomain of APP also has important roles, these studies demonstrate that the intracellular region of APP or APPL is indispensable to these functions.

Many strains of AD model mice (I refer to these mice collectively as AD mice) have been developed by inducing expression of FAD-linked mutant APP and presenilin. In these mice with aging, there is a loss of neurons and synapses as well as development of APs accompanied by dystrophic neurites (DNs), which is reminiscent of AD. Hyperphosphorylated tau, which is a constituent of neurofibrillary tangles (NFTs), is accumulated in DNs in a variety of AD mice (Sasaguri et al., 2017). At a young age, AD mice present network abnormalities. In a functional magnetic resonance imaging (fMRI) study, hypersynchrony of the default mode network (DMN)-like network is observed at the pre-plaque stage in APP*^NL–F/NL–F^* knock-in mice (Shah et al., 2018). Conversely, at 7 months of age when Aβ deposition appears, the DMN-like network is hyposynchronous in these mice. This dynamic pattern of resting-state network hypersynchrony before Aβ deposition and subsequent hyposynchrony at a later age is consistent with other studies in AD mice, specifically in Tet-off APP (TG) mice (Ben-Nejma et al., 2019) and in APPswe/PS1dE9 mice (Bero et al., 2012). In addition, network hyperexcitability and seizure susceptibility are common manifestations in AD mice, preceding Aβ pathology in 3XTg-AD mice (Kazim et al., 2017) and in Tg2576 mice (Bezzina et al., 2015).

In mice expressing M146V PSEN1 mutant as well as presenilin knockout mice, exuberant neurite outgrowth and hippocampal axonal sprouting are seen (Deyts et al., 2016). These changes are abrogated when APP expression is absent. The level of CTF of APP increases significantly in these mice, which is believed to be a mechanism for aberrant plasticity (Deyts et al., 2016). Mutations in FAD linked PSEN1 impair its function as γ-secretase (Zhou et al., 2017), and then CTF accumulation occurs. It should be noted that an increase in the CTF level is commonly observed among mice expressing various PSEN1 mutants (Li et al., 2016), suggesting that aberrant plasticity is a common manifestation among the mutants. fMRI studies revealed hypersynchrony in key regions of the DMN and a decrease in DMN deactivation in children with FAD-linked E280A PSEN1 mutant when compared with non-carrier children (mean age of participants: 13 y) (Quiroz et al., 2015). The DMN is most active during inwardly oriented mental activity and deactivated during memory tasks. The degree of task induced DMN deactivation is correlated with cognitive performance (Sperling et al., 2010). The DMN is rather impaired in the course of disease onset (Chhatwal et al., 2013). Earlier hypersynchronization and subsequent decline are similarly observed in AD mice (Bero et al., 2012; Shah et al., 2018; Ben-Nejma et al., 2019). During a face-name associative encoding task, neurons in the hippocampus are hyperactivated in presymptomatic E280A PSEN1 mutation carriers (age: 33.7 y) (Quiroz et al., 2010). Epileptic activity is also prominent among FAD pedigrees harboring the mutation in PSEN1, PSEN2, or APP (Zarea et al., 2016). Gray matter volume increases in children with PSEN1 mutant (Quiroz et al., 2015). It is conceivable that the volume of gray matter reflects the number of synapses, because gray matter volume and synapse number are well correlated at puberty (Blakemore and Choudhury, 2006).

Altogether, these observations suggest that network abnormalities are commonly present from an early stage of life in FAD mutant carriers and in AD mice.

## The Mechanism of Network Abnormalities

Synchronous firing of large groups of neurons can be elicited by the enhancement of excitatory synaptic connections or loss of inhibitory GABAergic drive onto principal neurons. Subgroups of GABAergic interneurons generate oscillatory rhythms through controlling the spike timing in neighboring principal neurons. Network abnormalities are often caused by synaptic remodeling of these inhibitory/excitatory neuronal circuits. APP and presenilin play important roles in synaptic plasticity, implying that network abnormalities seen in FAD patients and AD mice (abnormal DMN, decreased deactivation of DMN, seizure-like hyperexcitation and hippocampal hyperactivation during memory task) may result from aberrant plasticity caused by abnormal functions of these mutants. Indeed, hyperconnectivity and hyperexcitability of principal neurons have been observed at a young age in AD mice. In APP*^NL–F/NL–F^* knock-in mice, pyramidal neurons show persistent hyperexcitation by imbalance of excitation and inhibition (E/I), which originally occurs in the entorhinal cortex at 1-2 months of age (Petrache et al., 2019). In these mice, the function of parvalbumin-positive interneurons (PV cells) is impaired in the entorhinal cortex, and a network-driven component contributes to the E/I imbalance (Petrache et al., 2019). APP is highly expressed in GABAergic interneurons, especially in PV cells (Rice et al., 2020), enhances inhibitory tone to granule cells by regulating the formation of GABAergic synapses onto these cells (Wang et al., 2014), implying that dysregulation of GABAergic synapses caused by the mutant APP-induced disruption of PV cell microcircuitry leads to the E/I imbalance and hyperexcitation. In APPswe/PS1dE9 mice, at postnatal day 21, an increase in the number of medial apical synapses (CA3-CA1 connections) is seen in CA1 pyramidal neurons (Ray et al., 2020). Noting that the number of synapses simultaneously decreases at the distal apical tuft, the site for entorhinal inputs, an aberrant increase in the number of excitatory CA3-CA1 connections may reflect compensatory reweighting of synaptic connections. In aged transgenic mice (13-14 months), the number of synapses decreases uniformly across all dendritic and somatic compartments (Ray et al., 2020). Similarly, in Tg2576 mice, spine densities in hippocampal CA1 and cortical pyramidal neurons increase at 1 month of age before Aβ pathology and later decrease at 12 months of age (Lee et al., 2010).

PV cells are involved in forming gamma oscillations. Increasing the firing rate of PV cells enhances the power of gamma oscillations (Sohal et al., 2009). Conversely, impairment of PV cells is associated with aberrant hypersynchronization such as occurs in schizophrenia and epilepsy (Freund and Katona, 2007). In hAPPJ20 mice, it is demonstrated that functional deficit of PV cells causes network hypersynchrony during reduced gamma oscillatory activity (Verret et al., 2012). In APPswe/PS1dE9 mice, hippocampal PV cells are hyperexcitable at 4 months of age, but rather hypoexcitable at 6 months of age (Hijazi et al., 2020). It was also reported that the electroencephalogram power in gamma-band oscillations increases in the frontal cortex and the thalamus of APPswe/PS1dE9 mice at 16-17 weeks of age (Gurevicius et al., 2013). The pattern of early hyperexcitation and subsequent decline in PV cell activity is correlated with early network hypersynchrony and hyposynchrony at later age (Bero et al., 2012; Shah et al., 2018). It is proposed that a coupled set of local gamma oscillations at multiple brain regions leads to long-distance synchrony of the resting-state network (Cabral et al., 2014; Koelewijn et al., 2019). In patients with cingulate gyrus epilepsy, gamma oscillations may be a key contributor to DMN connectivity (Leng et al., 2020), and transient suppression of gamma oscillations is associated with DMN deactivation (Ossandón et al., 2011), implying that PV cells may regulate DMN activity through controling local gamma oscillations. The number of PV cells increases (Verdaguer et al., 2015) or decreases (Petrache et al., 2019) depending on the strain of AD mice. Similarly, the number of PV cells increases in the rat kindling model (Kamphuis et al., 1989), but decreases in the pilocarpine-induced epileptic model (Kobayashi and Buckmaster, 2003).

Differential phenotypes among AD mice may be due to distinct patterns and levels of transgene expression. Dysregulation of GABAergic synapses, increased spine density in principal neurons and increased/dysregulated gamma oscillations are differentially seen among AD mice, but all can be the molecular basis of network abnormalities. Results also depend on the age of examined mice. As is well known, hyperexcitability in principal neurons causes neuronal impairment and neuron loss, which eventually lead to hypoactivity of the neuronal network. As a compensatory response for hyperexcitation, remodeling of inhibitory circuits also occurs in AD mice (Palop et al., 2007). PV cells express α-Amino-3-hydroxy-5-methyl-4-isoxazolepropionic Acid (AMPA) receptors and are vulnerable to glutamate hyperexcitability (Moga et al., 2002). Increase or decrease in the number/activity of PV cells may depend on how inhibitory PV cell microcircuitry is remodeled.

Direct soluble Aβ administration into the hippocampus of mice can induce neuronal hyperexcitability (Busche et al., 2012). Soluble Aβ triggers hyperexcitation by suppressing glutamate reuptake (Zott et al., 2019), but paradoxically Aβ reduces glutamatergic transmission at the synaptic level (Kamenetz et al., 2003; Snyder et al., 2005). Aβ increases hippocampal PV cell excitability but not pyramidal neuron excitability (Hijazi et al., 2020). On the contrary, Aβ disrupts hippocampal gamma oscillations (Kurudenkandy et al., 2014). It is suggested that Aβ monomers, oligomers, fibrils, and APs behave differentially, by which multifunctional actions of Aβ are understood. Nonetheless, it is not conclusive that Aβ triggers the network abnormalities which are seen earlier than Aβ deposition. All in all, it can be proposed that network abnormalities seen at early stage of life results from FAD mutant-driven aberrant synaptic plasticity. The mechanism of how aberrant synaptic plasticity causes AD will be discussed later in this paper.

Individuals with Down’s syndrome develop AD pathology. Findings of different segmental duplications of chromosome 21 subregions in Down’s syndrome conclusively show that lifelong overexpression of wild-type APP causes AD (Selkoe and Hardy, 2016). If a hypothesis based on synaptic plasticity is correct, it follows that the cause of AD in Down’s syndrome is hyperfunction of APP. Nonetheless, it is uncertain whether the functional abnormality of FAD mutants can be simply described as excessive. In this paper, the word “aberrant” is preferably used to explain the pathogenesis of FAD.

## Apolipoprotein E ε4 and Sporadic Alzheimer’s Disease

Apolipoprotein E (APOE) has three isoforms, APOE ε2, 3, and 4, and individuals with APOE ε4 are more likely to develop AD. Thus, APOE ε4 is the genetic risk factor for sporadic Alzheimer’s disease (SAD) (Farrer et al., 1997). Numerous reports have indicated the roles of APOE in AD pathogenesis, but the underlying mechanism by which APOE ε4 increases the risk of AD is not fully understood. In mouse studies, the effects of APOE ε4 on neuronal plasticity are inconsistent. Both negative and beneficial effects have been associated with APOE ε4 (Kim et al., 2014). More essentially, it has been shown that inherent functional differences may exist between mouse and human APOE isoforms (Brendza et al., 2002), suggesting that much importance should be placed on studies in humans. In fMRI studies on healthy young adults, network abnormalities are commonly observed in APOE ε4 carriers. Just like individuals with presenilin mutant, DMN enhancement (mean age of participants: 28.4 y) (Filippini et al., 2009), (24 y) (Su et al., 2015), decreased DMN inactivation (19.7 y) (Shine et al., 2015) and increased hippocampal activation during memory tasks (23.2 y) (Dennis et al., 2010), (28.4 y) (Filippini et al., 2009) are observed in APOE ε4 carriers when compared with non-carriers. Hyperactivity in gamma-band oscillations is also seen in young APOE ε4 carriers (25.2 y) (Koelewijn et al., 2019). Alterations of DMN activity precede abnormalities in brain structure, blood flow, and memory performance (Filippini et al., 2009; Su et al., 2015), and probably Aβ deposition (Shine et al., 2015). According to studies in human derived neuronal cultures (Huang et al., 2017, 2019; Lin et al., 2018), APOE modulates APP expression (Huang et al., 2017, 2019) or metabolism (Lin et al., 2018), and promotes synaptic plasticity, which is substantiated by the number of synapses (Lin et al., 2018; Huang et al., 2019). The consensus of these reports is that APOE ε4 has the strongest effects on APP expression or metabolism, and synaptic plasticity, which is in line with neuronal network enhancement seen in young APOE ε4 carriers. Again, the DMN, once enhanced in young APOE ε4 carriers, is decreased in older carriers (Badhwar et al., 2017).

## Excessive and Maladaptive Synaptic Plasticity With Aging

It has been reported that during memory tasks, the activity of neurons in the medial temporal lobe or the hippocampus is greater in patients with early-stage mild cognitive impairment (MCI) than in healthy subjects, and that greater activation of neurons is associated with poorer performance (Dickerson et al., 2004; Yassa et al., 2010; Corriveau-Lecavalier et al., 2021a), indicating that such hyperactivation is detrimental to brain function. In fact, the drug levetiracetam ameliorates the lowered memory performance as well as hyperactive state in the hippocampus of MCI patients (Bakker et al., 2012, 2015). Aβ induces neuronal hyperexcitability in the hippocampus and hyperactive neurons are found preferentially (Busche et al., 2012) or exclusively (Busche et al., 2008) near the APs in AD mice. Thus, hyperactivation seen in the hippocampus of early MCI may simply reflect Aβ pathology. However, hippocampal hyperactivation during memory task is already detectable in healthy young APOE ε4 carriers who are unlikely to have Aβ deposition (Filippini et al., 2009; Dennis et al., 2010). Besides, hyperactivation is task-dependent, implying that it is associated with altered patterns of functional connectivity (Corriveau-Lecavalier et al., 2021a). Although remodeling of the neuronal network compensates for loss of brain function which declines with aging, it has also become evident that plastic remodeling is sometimes detrimental. Namely, greater activation of neurons in the newly constructed neural network in older adults is found to be correlated with poorer, not better task performance (Grady, 2012). As aging increases the demand for neuronal plastic remodeling, it is a tall order to fulfill it perpetually. Errors may arise during this process. If plastic remodeling, which is basically a compensatory mechanism for overcoming age-related functional decline, occurs excessively, it may lead to maladaptive, neural over-recruitment.

The activity of neurons in the hippocampus increases in early MCI and then decreases as the disease progresses. The shape of this temporal change in neuronal activity is described by the inverse U-shape model (Zott et al., 2018; Corriveau-Lecavalier et al., 2021b). If the activity of neurons increases in early MCI, how will cognitive function change thereafter? Follow-up studies for a period of 2 years or more demonstrate that cognitive function declines significantly and the rate of AD development increases when the baseline neuronal activity is greater (Dickerson et al., 2004; Dickerson and Sperling, 2008; Miller et al., 2008; O’Brien et al., 2010; Huijbers et al., 2015).

## The Function of TREM2 and Its Implication for Alzheimer’s Disease

A closer look at SAD risk genes found by genome wide association studies highlights the link between microglial cells and AD. Of particular interest is triggering receptor expressed on myeloid cells-2 (TREM2) (Guerreiro et al., 2013; Jonsson et al., 2013), a gene expressed in microglial cells. The R47H mutation in TREM2 has a similar risk as APOE ε4. Functional studies revealed that TREM2 variants related to AD reduce function, suggesting that the functional deficit of TREM2 increases the risk of AD (Song et al., 2018). It has been reported that in AD mice, APs are morphologically less compact and more damaging to surrounding neurites when TREM2 function is impaired (Wang et al., 2016; Yuan et al., 2016). In these papers, it is proposed that TREM2 has the function of protecting neurites from the toxicity of Aβ by wrapping Aβ compactly to block contact with neurites. Nonetheless, the assumption that the toxicity of Aβ causes neurodegeneration is not in accord with the fact that in rare cases DNs are present outside of APs (Blazquez-Llorca et al., 2017).

Staining with anti-GAP-43 antibody has shown that DNs associated with APs form growth cones with abnormal extension of neurites (Masliah et al., 1991). This pathological feature is called aberrant sprouting. GAP-43 is also present in DNs in AD mice. In APP23 mice, AP-associated DNs, which are identified as entorhinal axons or commissural axons, extend beyond boundaries and ectopically invade the inner molecular layer or the outer molecular layer of the dentate gyrus, respectively (Phinney et al., 1999). Although Aβ may not only damage neurites but also attract neurite outgrowth beyond normal boundaries, an alternative explanation is that aberrant plasticity caused by the mutant APP leads to axon terminal invasion of the ectopic region, and neurodegeneration itself is caused by the mutant APP-driven aberrant plasticity.

The long-known function of microglial cells is phagocytosis, but this classic notion has already been overturned (Andoh and Koyama, 2021). They act on neurons and play important roles in modulating neuronal functions, one of which is neuronal plasticity. They are engaged in several processes of synaptic plasticity, such as synapse formation, maturation, and elimination (pruning) (Andoh and Koyama, 2021). TREM2 plays an important role in the dynamics of synapse formation. In mice lacking TREM2, an increased number of synapses is observed in both histological and biochemical studies (Filipello et al., 2018; Qu and Li, 2020). The increased number of synapses may be due to impaired synaptic elimination (Filipello et al., 2018). Thus, it is plausible that TREM2 deficiency exacerbated neurite abnormalities in AD mice by leading to more prominent hyperconnectivity when the mutant APP drives synaptic remodeling aberrantly. It is also suggested that the synaptic maturation process is accelerated in mice deficient in TREM2 signaling (Andoh and Koyama, 2021). Further investigations are needed to elucidate the process of synaptic plasticity and the involvement of TREM2 especially at the aged stage, and thereby provide important clues to AD pathogenesis.

## Conclusion

AD is pathologically characterized by the existence of APs and NFTs, and symptoms of AD typically begin with mild memory loss and eventually lead to loss of the ability to carry on a conversation and respond to the environment. This sequence of clinical manifestations is consistent with the anatomic progression of the neurodegeneration. Lesions of AD begin in the hippocampus and spread to the temporal, frontal, and parietal lobes within the cerebral cortex. Numerous studies have been attempted to link AD-related molecules to the pathogenesis of AD: APOE ε4-associated mechanisms (Yamazaki et al., 2019) such as Aβ clearance and aggregation, cerebral energy metabolism, neuroinflammation, neurovascular function, and synaptic plasticity, and presenilin-related ones such as Aβ production (Selkoe and Hardy, 2016), calcium homeostasis (Honarnejad and Herms, 2012) and neurogenesis (Hernandez-Sapiens et al., 2022). Such heterogeneous and multiple mechanistic pathways may work cumulatively over a lifetime to increase an individual’s risk of AD. Nonetheless, the pathogenesis hypothesis needs to make logical connections with several confirmed findings, that is, the existence of both APs and NFTs, anatomical characteristics of neurodegeneration, and the similarity in pathology between FAD and SAD. The amyloid hypothesis has long been at the center of discussions. Aβ is believed to be toxic to neurons and have various mechanisms of action. It is also suggested that Aβ triggers tau aggregation and that the tau aggregate becomes a seed for further aggregation, leading to tau aggregate spread along neurons that are anatomically connected (Silva and Haggarty, 2020). Although this notion is not conclusive because of lack of evidence for the existence of a seed-competent form of extracellular tau and the mechanism of transcellular propagation (Silva and Haggarty, 2020), it can bridge the gap between APs and NFTs and provide a possible explanation for how the pathology spreads anatomically.

**FIGURE 1:**
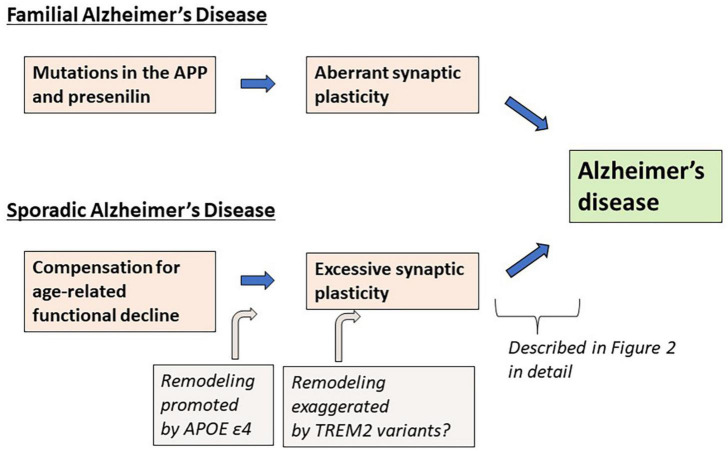
Summary of the synaptic plasticity hypothesis of Alzheimer’s disease.

**FIGURE 2:**
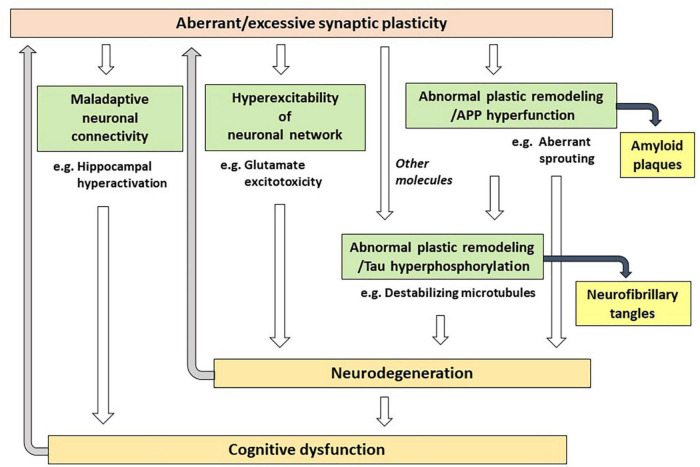
Possible mechanisms of how aberrant/excessive plasticity causes cognitive dysfunction in Alzheimer’s disease.

As an alternative to the Aβ hypothesis, in this paper, I propose that excessive (or aberrant) and maladaptive synaptic plasticity is the cause of AD (Figure 1). Previously, plasticity failure was proposed as a cause of AD (Mesulam, 1999). In this hypothesis, AD results if the demand for plastic remodeling exceeds the biological capacity to fulfill it. The author explains that FAD causing mutations of APP increase the demand for plastic remodeling by shifting the balance of its processing toward more toxic form of Aβ. Another example is the malignant synaptic growth hypothesis, which suggests that AD develops if the positive feedback mechanism during synaptic modification is dysregulated (Newman et al., 2012). The authors suggest that Aβ prevents neurons from malignant synaptic growth by impairing the function of plasticity-related synaptic molecules and that FAD-linked mutations produce types of Aβ which have a weaker neuroprotective effect against it. Network abnormalities have also been discussed as potential mechanisms of cognitive dysfunction in AD (Palop and Mucke, 2016; Harris et al., 2020). In these papers, Aβ is considered to be a central molecule causing network abnormalities. In contrast to the previous discussions, hypothesis proposed here states that excessive/aberrant synaptic plasticity is a root cause for cognitive dysfunction in AD and that cognitive dysfunction is developed through maladaptive neuronal connections, hyperexcitability of neuronal network and abnormal process of synaptic remodeling (Figure 2). APP is a key player in synaptic plasticity, and in FAD, the mutant APP or presenilin leads to aberrant plasticity through altered APP metabolism and function, which initially manifest as neuronal network abnormalities. Such aberrant and maladaptive synaptic plasticity is burdensome on neurons and renders them vulnerable. Hyperexcitability of neuronal network leads to glutamate excitotoxicity that has been hypothesized to play a role in the progressive neuronal loss that underlies AD (Rogawski and Wenk, 2003). It was also reported that neurons enriched with processes related to remodeling connections with adjacent neurons are vulnerable to AD (Roussarie et al., 2020). Tau is a key regulator for microtubule dynamics, which is fundamental in the formation and remodeling of synaptic structures. Phosphorylation of tau reduces its binding affinity to microtubules, by which tau regulates the stability of microtubule bundles. Accordingly, errors during burdensome bursts of exuberant plastic remodeling may cumulatively disturb the dynamics of microtubule formation. If tau is hyperphosphorylated concomitantly, it no longer can bind to microtubules allowing neurodegeneration to progress and hyperphosphorylated tau to accumulate. Thus, a perturbation of plasticity-associated activities stemming from functional abnormalities of the mutant APP or presenilin may lie at the heart of FAD pathogenesis. As described previously (Mesulam, 1999), it is conceivable that two pathological hallmarks of AD, APs and NFTs, represent byproducts of excessive/aberrant synaptic plasticity seen in the AD brain. If APs and NFTs are byproducts of excessive/aberrant synaptic plasticity, distinctive topographic distributions of APs and NFTs and individually variable patterns of them can be explained by the likelihood that the pathological appearances depend on several independent factors, such as intrinsic neuronal properties, properties of neighboring neurons and glial cells, the extracellular environments and how excessive/aberrant synaptic plasticity takes place. The baseline activities for plastic remodeling are rising with aging, in which plasticity-related molecules other than APP come to participate. These molecules like Nerve Growth Factor are known to upregulate the expression of tau and favors its phosphorylation (Mesulam, 1999), suggesting that they may also be involved in the degenerative process by accelerating hyperphosphorylation of tau (Figure 2).

AD pathology starts in a region of high plastic remodeling activity and progresses to regions of descending order of such activity (the entorhinal cortex and the hippocampus > the association neocortex > primary sensory-motor areas) (Arendt et al., 1998). The entorhinal cortex is the area of the brain in which AD pathology is first detectable in old age in individuals with or without MCI and AD. A compensatory response in the hippocampus is triggered by the damage in the entorhinal cortex (Geddes et al., 1985; Hyman et al., 1987). If plastic remodeling occurs excessively and maladaptively in the hippocampus, it may lead to the cognitive decline significantly and the rate of AD development may increase (Dickerson et al., 2004; Dickerson and Sperling, 2008; Miller et al., 2008; O’Brien et al., 2010; Huijbers et al., 2015). Among isoforms, APOE ε4, a genetic risk factor of SAD, exercises the strongest plasticity-promoting effect through an APP-related mechanism (Huang et al., 2017, 2019; Lin et al., 2018). The magnitude and the extent of brain activation during memory tasks in regions affected by AD including the left hippocampus, parietal, and prefrontal regions, are greater among APOE ε4 carriers than among APOE ε3 carriers (Bookheimer et al., 2000). If one has this risk factor, plastic remodeling which occurs with aging tends to become excessive and the risk of SAD may increase. Hyperactivation of neuronal networks seems to occur at different times for different brain regions. Task-related parietal hyperactivation is seen in individuals with late-stage MCI who already show hippocampal hypoactivation (Corriveau-Lecavalier et al., 2019). Excessive plasticity seems to be involved not only in disease onset but also in disease progression. AD patients are at risk for accelerated cognitive decline, if they show epileptiform activity, which is an indicator of network hypersynchrony (Vossel et al., 2016; Horvath et al., 2021).

As discussed in this paper, decades of research do not necessarily support only the amyloid hypothesis, but also can be utilized to hypothesize excessive/aberrant and maladaptive synaptic plasticity as the cause of AD. If this hypothesis is correct, an important goal aimed at delaying the onset of AD and slowing or halting the disease progression is to find ways to adaptively regulate synaptic remodeling without interfering with necessary changes. This requires an understanding of the fundamental mechanism of synapse dynamics, and the characteristics of the stage of synaptic plasticity (formation, maturation, or elimination) at which a person developing AD is affected. Because of heterogeneity between individuals, identifying the stage of synaptic plasticity at which individuals are prone to error is a prerequisite for providing each person the appropriate intervention.

## Clinical Application

Neuronal synchrony regulates the functional state of brain network and supports cognition. Because the network activities are altered before the clinical onset of AD, therapeutic interventions which counteract such network abnormalities may be able to ameliorate the cognitive dysfunction seen in persons with MCI and AD. However, this strategy of therapeutic intervention may only provide symptomatic effects and for the disease modification, it may be necessary to inhibit excessive synaptic remodeling, which is the basis of network abnormalities. Interestingly, levetiracetam attenuates expression of genes known to regulate synaptic remodeling (Christensen et al., 2010), suggesting that it does not only improve cognitive performance of MCI patients (Bakker et al., 2012, 2015), but can also palliates excessive synaptic plasticity and then prevent or delay disease progression.

The use of fMRI may allow early detection of hyperactive state in the hippocampus and the presence of excessive synaptic remodeling in MCI patients. However, it may be difficult to utilize fMRI as a biomarker because of its cost and scalability as well as the need for validation and normalization of the procedure. Recent progress has been made in use of blood biomarkers for the early recognition of persons at high risk for development of AD, especially measurement of phosphorylated tau (P-tau) species such as P-tau181, P-tau217, and P-tau231 (Verde, 2022). The gradual rise in plasma levels of P-tau species may be able to predict the age at which a person develops clinically recognized AD (Verde, 2022). When synaptic remodeling takes place, tau is phosphorylated and its binding affinity to microtubules is reduced, implying that an increase in the P-tau levels may be a sign of a perturbation of excessive plasticity-associated activities. Thus, the plasma level of P-tau species may be able to identify persons who may benefit from early therapeutic intervention.

## Data Availability Statement

The original contributions presented in this study are included in the article/supplementary material, further inquiries can be directed to the corresponding author.

## Author Contributions

SK contributed to the conceptualization and wrote the manuscript.

## Conflict of Interest

SK was employed by Sompo Care Inc.

## Publisher’s Note

All claims expressed in this article are solely those of the authors and do not necessarily represent those of their affiliated organizations, or those of the publisher, the editors and the reviewers. Any product that may be evaluated in this article, or claim that may be made by its manufacturer, is not guaranteed or endorsed by the publisher.
